# The Anti-Apoptotic Effect of ASC-Exosomes in an In Vitro ALS Model and Their Proteomic Analysis

**DOI:** 10.3390/cells8091087

**Published:** 2019-09-14

**Authors:** Roberta Bonafede, Jessica Brandi, Marcello Manfredi, Ilaria Scambi, Lorenzo Schiaffino, Flavia Merigo, Ermanna Turano, Bruno Bonetti, Emilio Marengo, Daniela Cecconi, Raffaella Mariotti

**Affiliations:** 1 Department of Neurological, Biomedical and Movement Science, University of Verona, 37134 Verona, Italy; roberta.bonafede@univr.it (R.B.); ilaria.scambi@univr.it (I.S.); lorenzo.schiaffino@univr.it (L.S.); flavia.merigo@univr.it (F.M.); ermanna.turano@univr.it (E.T.); 2 Department of Biotechnology, University of Verona, 37134 Verona, Italy; jessica.brandi@univr.it; 3 Department of Translational Medicine, University of Piemonte Orientale, 28100 Novara, Italy; marcello.manfredi@uniupo.it; 4 Center for Translational Research on Autoimmune and Allergic Diseases, University of Piemonte Orientale, 15121 Alessandria, Italy; emilio.marengo@uniupo.it; 5 Neurology Unit, Azienda Ospedaliera Universitaria Integrata Verona, 37126 Verona, Italy; bruno.bonetti@univr.it; 6 Department of Sciences and Technological Innovation, University of Piemonte Orientale, 15121 Alessandria, Italy

**Keywords:** extracellular vesicles, proteomic profiling, mesenchymal stem cells, amyotrophic lateral sclerosis, apoptosis, therapy

## Abstract

Stem cell therapy represents a promising approach in the treatment of several neurodegenerative disorders, including amyotrophic lateral sclerosis (ALS). The beneficial effect of stem cells is exerted by paracrine mediators, as exosomes, suggesting a possible potential use of these extracellular vesicles as non-cell based therapy. We demonstrated that exosomes isolated from adipose stem cells (ASC) display a neuroprotective role in an in vitro model of ALS. Moreover, the internalization of ASC-exosomes by the cells was shown and the molecules and the mechanisms by which exosomes could exert their beneficial effect were addressed. We performed for the first time a comprehensive proteomic analysis of exosomes derived from murine ASC. We identified a total of 189 proteins and the shotgun proteomics analysis revealed that the exosomal proteins are mainly involved in cell adhesion and negative regulation of the apoptotic process. We correlated the protein content to the anti-apoptotic effect of exosomes observing a downregulation of pro-apoptotic proteins Bax and cleaved caspase-3 and upregulation of anti-apoptotic protein Bcl-2 α, in an in vitro model of ALS after cell treatment with exosomes. Overall, this study shows the neuroprotective effect of ASC-exosomes after their internalization and their global protein profile, that could be useful to understand how exosomes act, demonstrating that they can be employed as therapy in neurodegenerative diseases.

## 1. Introduction

Amyotrophic lateral sclerosis (ALS) is a fatal neurodegenerative disease characterized by the selective degeneration of upper and lower motoneurons. ALS is a multifactorial disease since several pathogenetic mechanisms lead to motoneuron degeneration [1]. Due to this pathogenetic complexity, to date, no satisfactory treatment is available to cure or improve the quality of life of patients. The great interest of mesenchymal stem cells (MSC) as a therapeutic strategy in neurodegenerative diseases is due to their capability to migrate to damaged tissue and differentiate, contributing to stimulating the reparative processes [2,3]. Among the different sources of MSC, adipose stem cells (ASC) are easily available from liposuction, representing an interesting candidate in view to autologous transplantation [4]. Despite several preclinical and clinical applications provide evidence concerning the therapeutic potential of MSC in ALS, only a small number of transplanted cells reach the target tissue and differentiate [5,6]. To explain the therapeutic efficacy of MSC although the lack of cell engraftment, recent studies attribute the effect of stem cells to secretion of paracrine factors and to the release of extracellular vesicles [4,7]. This hypothesis provides the basis for a non-cell-based therapy for the treatment of different neurodegenerative diseases, including ALS, that could avoid the unpredictable consequences of cell therapy.

Exosomes are a group of extracellular vesicles 30 to 150 nm in diameter released by most cell types and can be taken up by recipient cells, covering an important role in intercellular communication [8]. Concerning the possible therapeutic use of exosomes isolated from MSC in neurodegenerative diseases, it has been shown that these vesicles stimulate nerve regeneration, remyelination, synaptic plasticity and neuronal protection [9,10,11]. Moreover, it has been reported that exosomes can protect dopaminergic neurons from neurodegeneration through the reduction of reactive oxygen species and apoptosis [12]. Farinazzo and colleagues demonstrated that, after oxidative stress, exosomes protect neuroblastoma cells and primary murine hippocampal neurons [13]. We here confirmed our previous data demonstrating the neuroprotective effect of exosomes isolated from ASC (ASC-exosomes) in an in vitro model of ALS [14]. In this previous study, we used the motoneuron-like cell line NSC-34 transfected with a vector expressing mutated human *SOD1* gene (the first gene identified to be related with ALS). The mutations studied were *G93A*, *G37R* and *A4V*, and we showed that, in all cases, exosomes protect the cells from oxidative stress. Since the content of exosomes (in terms of lipids, proteins, and RNAs) is related to the origin cell, vesicles obtained from a different source could exert a different effect. The importance of knowing the content of the exosomes could lead to a better understanding of their mechanism of action on recipient cells in view of a possible their therapeutic use.

The objective of this study was to correlate the anti-apoptotic effect of ASC-exosomes after their internalization in the in vitro model of ALS with their comprehensive proteomic analysis. We used the NSC34 cells transfected with a vector expressing human *SOD1(G93A)* gene, since *G93A* mutation is the most commonly used to generate transgenic ALS models. We demonstrate that the biological effect on NSC-34(*G93A*) cells was determined by the uptake of ASC-exosomes. To this purpose, we used a method to label exosomes with ultra-small superparamagnetic iron oxide nanoparticles (USPIO, 4–6 nm), that allow the detection of labelled exosomes (exosomes-USPIO) in the cells [15]. Moreover, the shotgun proteomics analysis performed to assess the content of the vesicles revealed that the exosomal proteins are mainly involved in cell adhesion and negative regulation of the apoptotic process. In relation to this, we observed an upregulation of anti-apoptotic protein Bcl-2 α and downregulation of pro-apoptotic proteins Bax and cleaved caspase-3 after treating cells with exosomes, correlating the protein content to the neuroprotective effect on NSC-34 cells. These findings shed light on how these exosomes mediate their beneficial effects and provide for the first time a rationale for their use in treating neurodegenerative diseases.

## 2. Material and Methods

### 2.1. ASC and NSC-34 Cell Cultures

The ASC were isolated from inguinal adipose tissues of 8/12 week-old C57BL/6J mice (Charles River, Italy), as previously described [16]. The experiments were performed with the approval of the Italian Minister of Health, following the NIH guide for the use and the care of laboratory animals, in accordance with the current European Communities Council Directive (2010/63/EEC) and conformity to the international guidelines, minimizing the number of animals used and avoiding their sufferance. The mice were maintained under controlled environmental parameters with food and water ad libitum, with 12 h of light and dark cycle. Briefly, the tissue was incubated in Hank’s Balanced Salt Solution (HBSS, Life Technologies, Milan, Italy) with collagenase type I (Life Technologies) and bovine serum albumin (BSA, AppliChem, Euroclone, Milan, Italy). The stromal vascular fraction (SVF) obtained after centrifugation was resuspended in NH_4_Cl, centrifuged and filtered through a 40-μm nylon mesh to remove cell debris. ASC were cultured in Dulbecco’s Modified Eagle Medium (DMEM) with 10% heat-inactivated Fetal Bovine Serum (FBS), 100 U/mL penicillin and 100 μg/mL streptomycin (all from GIBCOLife Technologies, Milan, Italy) and incubated at 37 °C in a 5% CO_2_ atmosphere. Murine ASC were recognized by immunophenotype using monoclonal antibodies, as previously described [16]. These cells were used to isolate exosomes.

The NSC-34 motoneuron-like cell line was purchased from CELLutions Biosystems Inc. (Burlington, ON, Canada). NSC-34 cells were transfected with the human *SOD1* gene (*hSOD1*) carrying *G93A* point mutation (NSC-34(*G93A*)), as previously described [14]. NSC-34 cells were cultured in DMEM with 10% heat-inactivated FBS, 100 U/mL penicillin and 100 μg/mL streptomycin and incubated at 37 °C in a 5% CO_2_ atmosphere. These cells were used to confirm the neuroprotective effect of exosomes, to validate effect of exosomal proteins involved in the apoptotic pathway and to determine the internalization of exosomes.

### 2.2. Expression Vectors and Generation of Tetracycline-Inducible Cells Overexpressing His-HA-SOD1(G93A)

The plasmid pcDNA3-*SOD1*(*G93A*), expressing the *hSOD1* gene containing the *G93A* mutation, was purchased from Addgene (Cambridge, MA, USA) and used as template to amplify by PCR the respective cDNA. Briefly, *hSOD1*(*G93A*) cDNA was amplified with PfuDNA polymerase (Stratagene) using the following primers containing Sgf I and Mlu I restriction sites: Fw 5′GAGGCGATCGCCGCGACGAAGGCCGTGTGCGTGCTG3′ (Sgf I); Rv 5′ GCGACGCGTTTATTGGGCGATCCCAATTACAC3′ (MluI). The amplified fragment was digested with Sgf I and MluI enzymes and cloned into the pCMV6-AN-His-HA plasmid (PS100017, OriGene, Rockville, MD, USA) to give the vector pCMV6-HIS-HA-SOD1(G93A), expressing the mutant *hSOD1* gene in fusion with an amino-terminal polyhistidine (His) tag and a hemagglutinin (HA) epitope.

To generate the lentiviral vectors for the conditional expression of *SOD1* mutants, the *hSOD1*(*G93A*) gene was excised from pCMV6-HIS-HA-SOD1(G93A) plasmid by using BamHI and XhoI enzymes and subcloned in the same sites of the vector pENTR1A (w48-1, Addgene). This vector was then recombined into pLenti CMV/TO Puro DEST (670-1, Addgene) using LR-Clonase (Life Technologies) to give a lentiviral vector expressing *hSOD1*(*G93A*) gene under the control of a doxycycline-inducible promoter [14].

To establish an inducible cell line overexpressing the *hSOD1*(*G93A*) mutant, NSC34 cells were first transduced with the pLentiCMV_TetR_Blast vector (716-1, Addgene), that constitutively expresses high levels of the tetracycline (Tet) repressor under the control of a CMV promoter, and selected for 7 days using 10 µg/mL Blasticidin (Sigma). After drug selection, the stable cells were infected with the lentiviral vectors expressing *hSOD1*(*G93A*) in the presence of 4 µg/mL polybrene. The selection of transduced cells was then conducted using 5 µg/mL puromycin. The expression of *hSOD1* mutants was induced by adding 2 µg/mL doxycycline (Clontech) to the culture medium for the last 48 h of culture. The efficiency of *SOD1* mutant induction was quantified with a high content imaging approach, as previously described [14].

### 2.3. ASC-Exosomes and Exosomes-USPIO Isolation

Exosomes were isolated from the culture medium of 1 × 10^7^ ASC. Murine ASC were cultured to confluence. To isolate exosomes from ASC cell culture conditioned medium and to avoid any contamination of shed membrane fragments and vesicles from serum, FBS deprivation for 48h was made. Cell culture supernatants were then collected and PureExo^®^ Exosome isolation kit (101Bio, Mountain View, CA, USA) was used for exosomes isolation, following the manufacturer’s protocol. The determination of the protein content of exosomes was determined by Bicinchoninic Protein Assay (BCA) method, using the manufacturer’s protocol (Thermo Scientific™ Pierce™ BCA™ Protein Assay). Moreover, the concentration of ASC-exosomes was assessed by NanoSight instrument (Izon Nanoparticle Tracking Analysis). The ASC-exosomes were used for their characterization by transmission electron microscopy (TEM) and western blot, for the proteomic analysis and for the evaluation of the neuroprotective effect in NSC-34 cells.

To obtain labelled ASC-exosomes, ASC (10^7^ cells) were incubated with 200 µg Fe/mL of ultra-small superparamagnetic iron oxide nanoparticles (USPIO, 5–7 nm) for 24 h, washed and deprived of FBS for 48 h to avoid any contamination of vesicles from serum. After deprivation, ASC supernatants were collected and exosomes-USPIO were isolated using PureExo^®^ Exosome isolation kit (101Bio, Mountain View, CA, USA). The determination of the protein content of exosomes was determined by the BCA method (Thermo Scientific™ Pierce™ BCA™ Protein Assay). The exosomes-USPIO can be detected by TEM, as previously reported [15]. The exosomes-USPIO were used to detect their internalization by the NSC-34(G93A) cells by TEM.

### 2.4. Electron Microscopy of ASC-Exosomes

Exosomes pellet was fixed in 2% glutaraldehyde in Sorensen buffer (pH 7.4) for 2 h, post-fixed in 1% osmium tetroxide (OsO_4_) in aqueous solution for 2 h, dehydrated in graded concentrations of acetone and embedded in Epon–Araldite mixture (Electron Microscopy Sciences, Fort Washington, PA, USA). The semithin sections (1 µm in thickness) were examined by light microscopy (Olympus BX51, Olympus Optical, Hamburg, Germany) and stained with toluidine blue. The ultrathin sections were cut at a 70 nm thickness, placed on Cu/Rh grids with Ultracut E (Reichert, Wien, Austria), and observed with TEM using a Morgagni 268D electron microscope (Philips).

### 2.5. Biochemical Characterization of ASC-Exosomes by Western Blot

Analysis of exosomes by immunoblotting was performed using standard protocols: Proteins were denatured, separated on 4–12% polyacrylamide gels, transferred onto a nitrocellulose membrane and probed with antibodies against heat shock protein 70 (HSP70, 1:100 Santa Cruz Biotechnology, sc-1060), and tetraspanins CD9 (1:100 MM2/57, Millipore CBL-162) and CD81 (1:100 Santa Cruz Biotechnology, sc-9158) followed by appropriate horseradish peroxidase (HRP) conjugated secondary antibodies against the primary antibody (all secondary antibodies were from Dako Agilent). ASC lysates were used as the positive control. The blots were then incubated with a chemiluminescent HRP substrate and detected with G:BOX F3 GeneSys (Syngene, UK).

### 2.6. Sample Preparation for Shotgun Proteomics

ASC-exosomes were collected and lysed in 1X PBS added with protease inhibitors cocktail 1X (Roche) and 1% sodium dodecyl sulphate (SDS) (Bio-Rad, Hercules, CA, USA). Protein extraction was performed by 5–6 cycles of sonication; then a four-fold volume of ice-cold acetone was added to samples and protein precipitation was conducted overnight at −20 °C. Samples were then centrifuged at 14,000× *g* for 10 min at 4 °C, and the pellet was resuspended in 100 mM NH_4_HCO_3_. Protein concentration was measured with BCA Protein Assay (Thermo Fisher Scientific) using bovine serum albumin as a standard. Before the shotgun proteomic analysis, proteins were digested as already described [17]. Briefly, 40 µg of proteins were reduced by using dithiothreitol and alkylated with iodoacetamide. Then, after dilution with 100 mM NH_4_HCO_3_ solution, 5 µg of trypsin (Promega, Sequence Grade) was added and digestion was performed overnight at 37 °C. Trypsin activity was stopped by adding 2 µl of neat formic acid and digests were dried by Speed Vacuum.

### 2.7. Mass Spectrometry Analysis

The digested samples were analyzed on a micro-LC (Eksigent Technologies, Dublin, CA, USA) interfaced to a 5600+ TripleTOF mass spectrometer system (AB Sciex, Concord, ON, Canada) equipped with a DuoSpray Ion Source and a CDS (calibrant delivery system). The LC column was a Halo Fused C18 with a pre-column ProteCol C18G. The mobile phase was a mixture of 0.1% (*v*/*v*) formic acid in water (A) and 0.1% (*v*/*v*) formic acid in acetonitrile (B), eluting at a flow-rate of 15 μL min^−1^ at an increasing concentration of solvent B from 2% to 86% in 17 min. The injection volume was 4 μL and the oven temperature was set at 40 °C. For identification purposes, the mass spectrometer analysis was performed using a mass range of 100–1500 Da (TOF scan with an accumulation time of 0.25 s), followed by a MS/MS product ion scan from 200 to 1250 Da (accumulation time of 5 ms) with the abundance threshold set at 30 cps (35 candidate ions can be monitored during every cycle). The ion source parameters in the electrospray positive mode were set as follows: Curtain gas (N_2_) at 25 psig, nebulizer gas GAS1 at 25 psig, and GAS2 at 20 psig, ion spray floating voltage (ISFV) at 5000 V, source temperature at 450 °C and declustering potential at 25 V.

The DDA (Data Dependent Analysis) files were searched using Protein Pilot software v. 4.2 (AB SCIEX, Concord, ON, Canada) and Mascot v. 2.4 (Matrix Science Inc., Boston, MA, USA). Trypsin as a digestion enzyme was specified for both software. For Mascot, we used two missed cleavages, the instrument was set to ESI-QUAD-TOF and the following modifications were specified for the search: Carbamidomethyl cysteine as a fixed modification and oxidized methionine as variable modification. A search tolerance of 0.08 Da was specified for the peptide mass tolerance, and 10 ppm for the MS/MS tolerance. The charges of the peptides to search for were set to 2+, 3+ and 4+, and the search was set on monoisotopic mass. The UniProt Swiss-Prot reviewed database containing mouse proteins (version 2015.20.07, containing 23,304 sequence entries, UniProt Consortium) was used and a target-decoy database search was performed. The false discovery rate (FDR) was fixed at 1% [18,19].

### 2.8. Bioinformatics Analysis

The identified exosomal proteins were subjected to bioinformatics analyses in order to: Investigate the presence of surface proteins, classify them on the basis of gene ontology, identify the molecular pathways in which they are involved, and highlight any protein–protein interaction networks. In particular, the detection of identified exosomal proteins located on the plasma membrane (which potentially can be shed and released to the extracellular space) was performed by TransMembrane prediction using hidden Markov models (TMHMM 2.0 server) (http://www.cbs.dtu.dk/services/TMHMM/) which predicts transmembrane helices [20]. Functional annotation of identified proteins was employed using the Database for Annotation, Visualization and Integrated Discovery (DAVID) (v6.8) (http://david.abcc.ncifcrf.gov/) [21] to identify gene ontology (GO) biological processes (GOBPs), molecular function (GOMFs) and cellular component (GOCCs). The GOBPs, GOMFs, and GOCCs enriched by the list of proteins were identified as the ones with *p*-value < 0.01 calculated by DAVID.

The Cytoscape (v3.5.0) ClueGO (v.2.5.0) plugin [22] was used to visualized enriched pathways associated with the Kyoto Encyclopaedia of Genes and Genome (KEGG) database. In brief, KEGG pathways were explored with medium specificity and a kappa score of 0.4. An enrichment/depletion method with a two-sided hypergeometric test was applied, correct with the Bonferroni step down for each p-value calculation. Enriched pathways with a *p*-value < 0.05 were considered significant.

Finally, in order to predict protein-protein interaction, the identified proteins of ASC-exosomes were analyzed using STRING software (http://string-db.org) [23]. STRING analysis was performed by setting the species under investigation (*Mus musculus*) with a medium confidence level (score 0.4); we retrieved interactions based on experimental and database knowledge, excluding all other prediction methods implemented in STRING (such as co-expression and text-mining). Additional white nodes and network depth were kept to the minimum value (1), to exclude as many false positive interactions as possible.

### 2.9. Immunoblotting of Akt and SOD1

Exosomes and ASC were subjected to immunoblotting analysis using the following protocol: Proteins were extracted in RIPA buffer (Sigma-Aldrich) containing protease inhibitor cocktail tablets 1X (Roche), sonicated 10 min and then lysates were clarified by centrifugation at 8000× *g* for 10 min at 4 °C. Supernatants were harvested and protein content was determined by BCA assay (Sigma-Aldrich). Samples were denatured, separated on 10–20% polyacrylamide gels, transferred onto a PVDF (polyvinylidene difluoride) membrane and probed with antibodies against phospho-Akt (Ser473) (1:1000, Cell Signalling Technologies; #9271), and *SOD1* (1:1000, NBP1-31204), followed by an appropriate HRP-conjugated secondary antibody against the primary antibody (Santa Cruz Biotechnology, sc-2005). ASC lysate was used as a positive control. The blots were then incubated with a chemiluminescent HRP substrate and detected with ChemiDoc MP imaging system (Bio-Rad).

### 2.10. SOD1 Zymogram Assay

ASC-exosomes and adipose serum-deprived stem cells were lysed in 1XPBS containing protease inhibitor cocktail tablets 1X (Roche) for four times at 25 Hz for 10 s. Samples (60 μg per lane) were loaded in a native buffer and separated on a 15% (*v*/*v*) non-denaturing polyacrylamide gel for 3 h at 40 mA at 4 °C. The gel was stained for 45 min in 50 mM potassium phosphate (KH_2_PO_4_) pH 7.4 containing 275 μg/mL nitro blue tetrazolium (NBT) (ThermoFisher), 65 μg/mL riboflavin (Sigma-Aldrich) and 3.2 μL/mL tetra methyl ethylene diamine (TEMED) (Sigma-Aldrich) at room temperature in the dark. Gel was then illuminated for 15 min until sufficient contrast between achromatic zones (dismutase activity) and blue background was achieved and then scanned for documentation. ASC cells extract was used as positive control. An identical gel was stained with Coomassie brilliant blue to verify the equal amount of protein extract loaded among the different samples.

### 2.11. NSC-34 Cell Treatment and Viability

The NSC-34(G93A) cells were seeded at a density of 2 × 10^4^ and exposed to H_2_O_2_ (100 µM) for 6 h with or without the presence of ASC-exosomes (0.2 µg/mL, corresponding to 6–8 × 10^5^ particles/mL) in the culture medium. The number of cells seeded, the quantity of exosomes able to protect the cells, the concentration of H_2_O_2_ used as pathological insult to induce apoptosis, and the time of H_2_O_2_ incubation with or without exosomes were identified in our previously study [14]. Cells (2 × 10^4^) without treatments were used as a basal condition. Cell viability and apoptotic cells were evaluated by cell counting after acridine orange/propidium iodide (AO/PI, both from Sigma-Aldrich) double staining. A total of 10 μL of AO/PI was added and spread by placing a coverslip over it. The apoptotic and live cells were visualized using an upright fluorescent microscope (DM6000B, Leica Microsystem) by their red or green fluorescence and their nuclear morphology. Each experiment was performed at least three times, and for each condition three replicates were performed. The number of stained cells were counted in 20 random field using the 20× objective. To evaluate the neuroprotective effect of ASC-exosomes on NSC-34(G93A) cells, the univariate analysis of variance (ANOVA) and Bonferroni post-hoc tests were performed to evaluate differences between the experimental conditions. Significance was accepted at *p* < 0.05.

### 2.12. Evaluation of Apoptotic Markers by Western Blot

The NSC-34(G93A) cells were exposed to H_2_O_2_ for 6 h with or without the presence of ASC-exosomes in the culture medium, as previously described [14]. A total of 2 × 10^4^ NSC-34(G93A) cells were used to perform western blot analysis. The western blot was performed by using pooled biological samples. Immunoblot was performed as previously described [24]. Briefly, after separation on 10–20% SDS-PAGE, proteins were electroblotted on a PVDF membrane and subjected to immunorevelation. Cleaved caspase 3 was detected by using anti-(cleaved) caspase 3 (1:300, Cell Signalling Technologies; #9661) and anti-rabbit secondary antibody-HRP conjugate (Santa Cruz Biotechnology, sc-2004); Bcl-2 α was investigated by using anti-Bcl-2 α (1:200, Biosource) primary antibodies and anti-rabbit secondary antibody-HRP conjugate (Santa Cruz Biotechnology, sc-2005) and Bax was detected by using anti-Bax (N20) (1:200; Santa Cruz Biotechnology sc-493) primary antibodies and anti-rabbit secondary antibody-HRP conjugate (Santa Cruz Biotechnology, sc-2005). The immunocomplexes were visualized by chemiluminescence using the ChemiDoc MP imaging system (Bio-Rad) and the intensity of the chemiluminescence response was measured by processing the image with Image Lab software (Bio-Rad).

### 2.13. Internalization of Exosomes-USPIO by NSC-34(G93A) Cells

To detect the internalization of ASC-exosomes by the cells, exosomes labelled with USPIO nanoparticles, that allowed the visualization by TEM, were used. The NSC-34(G93A) cells were seeded at a density of 2 × 10^4^ with the presence of exosomes-USPIO (0.2 µg/mL, corresponding to 6–8 × 10^5^ particles/mL) in the culture medium for 6 h. After the incubation time, the cells were washed with PBS, trypsinized and centrifuged. For ultrastructural morphology of cells, the pellet was fixed in 2% glutaraldehyde in Sorensen buffer (pH 7.4) for 2 h. The samples were post-fixed in 1% osmium tetroxide (OsO_4_) for 2 h, cut, dehydrated in graded concentrations of acetone and embedded in Epon-Araldite mixture (Electron Microscopy sciences, Fort Washington, PA, USA). The semithin sections (1 µm in thickness) were examined by light microscopy (Olympus BX51, Olympus Optical, Hamburg, Germany) and stained with toluidine blue. The ultrathin sections were cut at a 70 nm thickness, placed on Cu/Rh grids with Ultracut E (Reichert, Wien, Austria). TEM images were acquired with a Philips Morgagni TEM operating at 80kV and equipped with a Megaview II camera for digital image acquisition.

## 3. Results

### 3.1. Isolation and Characterization of ASC-Exosomes

**Figure 1:**
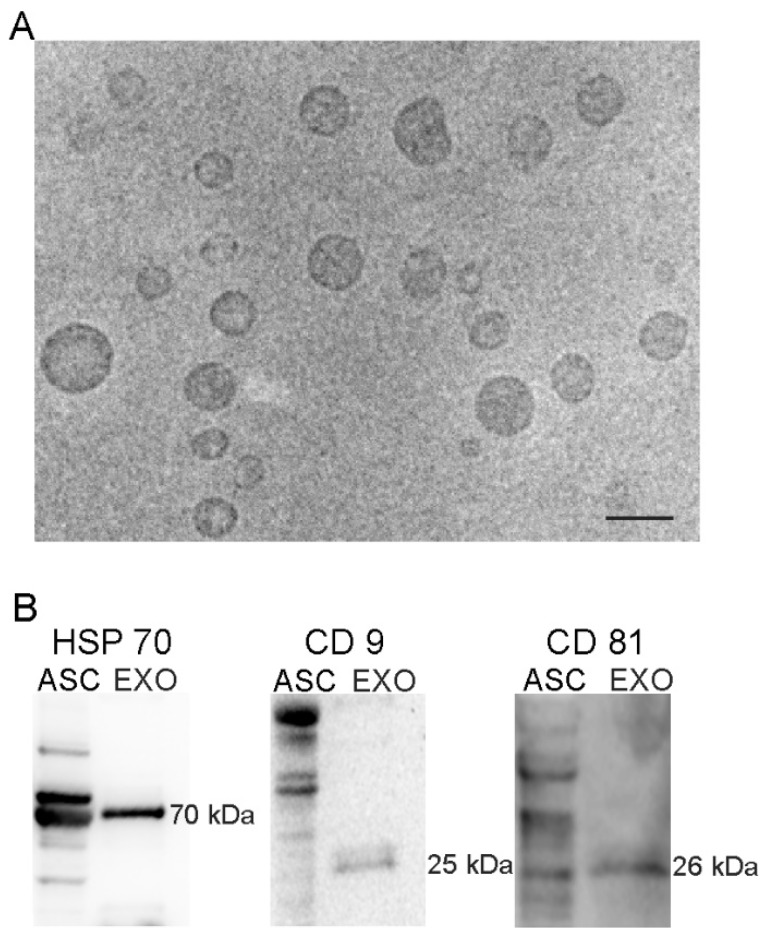
TEM and western blot analysis of adipose stem cells (ASC)-exosomes. Electron microscopy shows vesicles with characteristic morphology and size of exosomes. Scale bar, 100 nm (**A**). The blots show western blot detection of the expression of HSP70 (70 kDa), CD9 (25 kDa) and CD81 (26 kDa) in exosomes (EXO); ASC lysates (ASC) was used as positive control (**B**).

Exosomes were obtained with an exosomes isolation kit and their protein concentration was quantified. The yield for each isolation was about 100–150 µg of protein. The concentration of nanovesicles obtained by NanoSight were 6–8 × 10^8^ particles/mL. Ultrastructure analysis of the exosomes by TEM revealed round vesicles with lipid bilayers with a diameter of 50 to 150 nm (Figure 1A). Western blot analysis revealed specific exosomal markers including HSP70 (70 kDa), CD9 (25 kDa) and CD81 (26 kDa) (Figure 1B). These results confirm that size, morphology and the presence of specific protein markers are consistent with a previous report [25].

### 3.2. Proteomic Analysis of ASC-Exosomes and Annotations of Identified Proteins

The shotgun proteomic analysis of exosomes was performed to obtain the complete characterization of the proteome. We identified a total of 189 proteins with a peptide confidence cut-off of 99% (FDR < 1%). The identities of these proteins are presented in Supplementary Material Table S1.

After MS the identified exosomal proteins were analyzed using bioinformatics analysis. For each identified ASC-exosomes protein, the number of transmembrane regions was predicted using TMHMM. In particular, a total of 16 proteins were predicted to have transmembrane domains (TMDs), of which one has ten TMDs, i.e., ATPase class II type 9B, and all the others with one TMD; the remaining proteins were localized as outside (169) or inside the membrane [22] (Supplementary Material Table S2).

**Figure 2:**
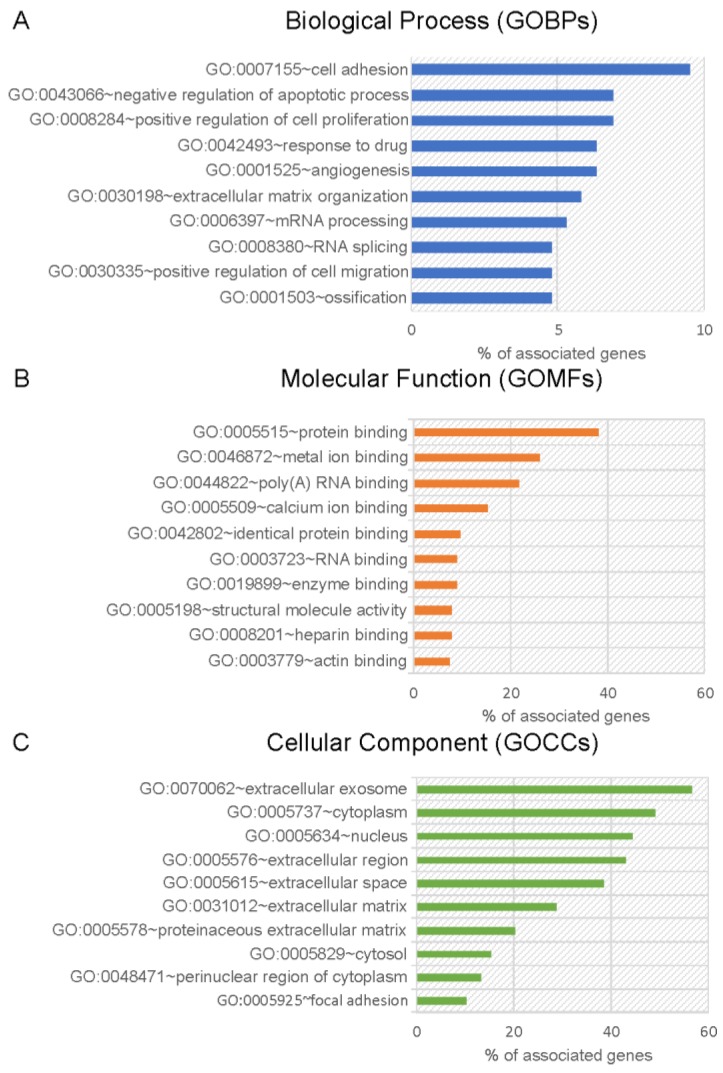
Gene ontology (GO) enrichment of the ASC-exosomes identified proteins according to Database for Annotation, Visualization and Integrated Discovery (DAVID) functional annotation. The top 10 enriched biological process (**A**) molecular function (**B**) and cellular component (**C**) are reported. The percentage represents the portion of the genes encoding the proteins with the corresponding gene ontology biological processes (GOBPs), gene ontology molecular functions (GOMFs) or gene ontology cellular components (GOCCs) in the ASC-exosomes proteins.

Functional annotation of ASC-exosomes proteome was examined using DAVID software by performing enrichment analysis of GOBPs, GOMFs, and GOCCs (Figure 2, and Supplementary Material Table S3). The 189 identified proteins have been associated with a total of 31 statistically significant (*p*-value < 0.01) GO terms for biological process. Interestingly, the most important significantly enriched GOBPs categories included cell adhesion (9.5%), negative regulation of apoptotic process (6.9%) and angiogenesis (6.3%) (Figure 2A). The most enriched GO terms for molecular functions were protein binding (40.7%), metal ion binding (24.9%), and poly(A) RNA binding (21.7%) (Figure 2B). Moreover, the enrichment analysis of GOCCs revealed that exosomal proteins were localized mainly into the extracellular exosome (i.e., vesicles released into the extracellular region) (52.9%), as well as the cytoplasm (47.6%) and extracellular region (41.8%) (Figure 2C).

**Figure 3:**
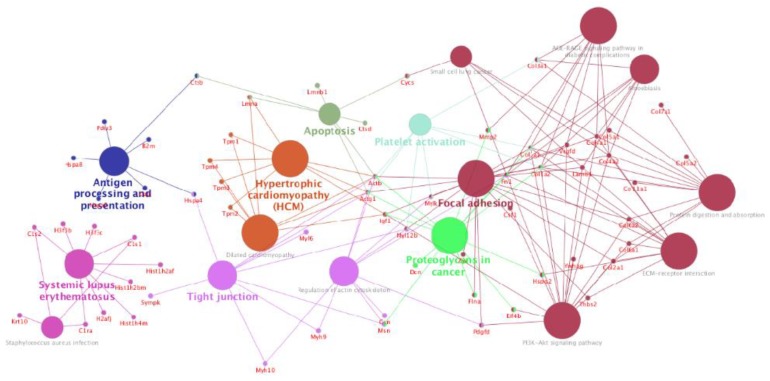
Cytoscape based ClueGo/CluePedia pathway analysis and visualization. Enriched pathways were obtained from the Kyoto Encyclopaedia of Genes and Genome (KEGG) database. Terms are grouped based on shared genes (kappa score) showed in different colors. The size of nodes indicated the degree of significance. The most significant term defined the name of the group.

To further explore the enriched pathways associated with ASC-exosomes proteins, we have imported the list of identified proteins in the ClueGO app for Cytoscape platform to create a network of the overrepresented GO terms. Only significant pathways or terms were presented by setting the statistical threshold (*p*-value < 0.05) and using the KEGG database as a reference (Supplementary Material Table S4). Multiple functions related to focal adhesion (such as Extracellular matrix receptor interaction, PI3K-Akt signaling pathway, protein digestion and adsorption), as well as to apoptosis, antigen processing and presentation, platelet activation, and proteoglycan in cancer were enriched for ASC-exosomes proteins (Figure 3).

**Figure 4:**
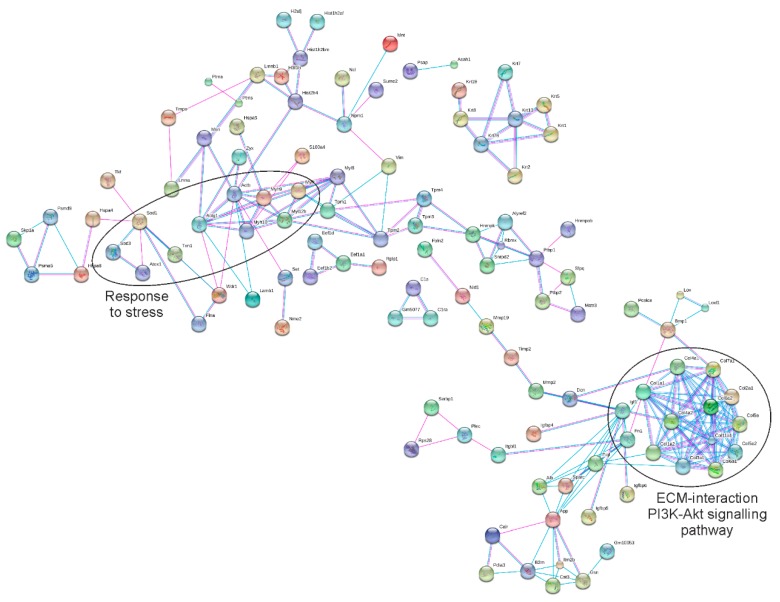
Protein network of identified ASC-exosomes proteins. Schematic view of known and predicted protein interactions according to the STRING database (v. 10). Each node represents a protein, and each edge represents an interaction. Only interactions with the medium confidence score (0.4) are shown. Interactions include physical and functional associations, showing the evidence view.

Finally, to investigate the interaction pattern of annotated proteins and to elucidate the physical interaction between them, we created a protein–protein interaction network of the ASC-exosomes proteome using STRING software. STRING analysis emphasized that ASC-exosomes proteins interact within the actin-myosin and collagen complexes, and showed functional relationship (i.e., contribute jointly to a specific biological function) regarding the mechanism of response to stress, PI3K-Akt signaling and interactions at extracellular matrix level (Figure 4). Concerning these pathways, several molecules could have a role to counteract neurodegenerative mechanisms. Through the proteomic analysis, we found some interesting proteins to counteract pathogenic mechanisms, as *SOD1*, that can destroy free superoxide radicals, the neuroprotective ribonuclease RNase 4, the insulin-like growth factors Igf1 and Akt, that play a role in inhibiting apoptosis (Supplementary Material Table S1, Figure 4). Since only the active form of proteins allows the correct function of the molecules, the validation of the enzymatically active form of the interested molecules was required. Among the interest proteins, RNase4 and Igf1 are already enzymatically active, while *SOD1* and Akt required some post-traductional modification to be activated. To this purpose, the presence of the active form in ASC-exosomes was validated.

### 3.3. Expression Profile of Phospho-Akt and SOD1 Proteins in ASC-Exosomes

It is known that, in ALS, the major mechanisms involved in motoneurons death are linked to oxidative stress and apoptosis. Since the proteomic analysis revealed the presence of proteins involved in these pathways, we focus attention on validating the presence of the active form of selected proteins (phospho-Akt for PI3K-Akt signaling pathway and *SOD1* for response to oxidative stress mechanism).

**Figure 5:**
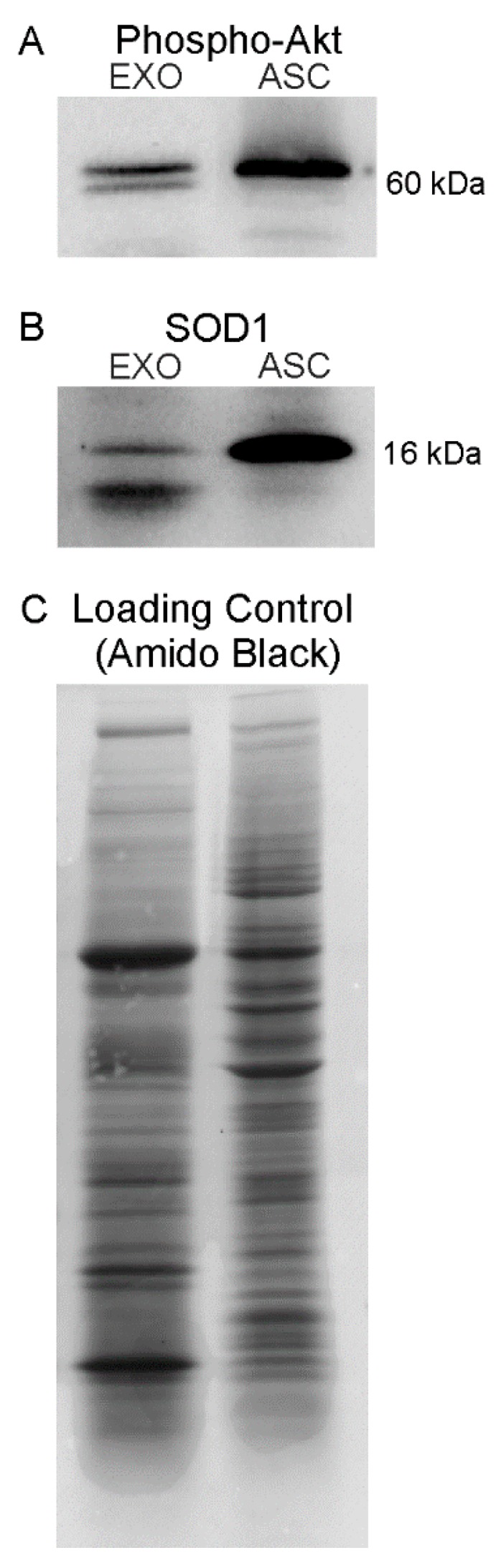
Western blot analysis of phospho-Akt and *SOD1* expression in ASC-exosomes and ASC. The blots show western blot detection of the expression of phospho-Akt (60 kDa) (**A**) and SOD1 (16 kDa) (**B**) in exosomes (EXO); ASC lysates were used as positive control. Amido Black staining was used as total loading control (**C**).

To investigate if an Akt protein is enzymatically active in ASC-exosomes, the immunoblot analysis of phospho-Akt (Ser473) was performed. As shows in Figure 5A, Akt was found active both in ASC and exosomes, thus confirming the presence of active proteins in ASC-exosomes. Notably, the multiple bands detected of phospho-Akt may represent the phosphorylated forms of the different Akt isoforms (i.e., Akt1, Akt2, and Akt3) which are all detected by the antibody.

Moreover, the presence of *SOD1* protein, previously detected by proteomic profiling of ASC-exosomes, was validated by western blot (Figure 5B). In addition, *SOD1* is observed as multiple bands in exosomes, these may represent spliced variants, truncated protein or post-translational modifications, which may be detected by the antibody used.

In order to assess if the SOD1 proteins contained in ASC-exosomes are enzymatically functional, we performed a zymogram assay. The native gel was stained using a solution of nitro blue tetrazolium and riboflavin. Riboflavin is a source of superoxide anions which interact with nitro blue tetrazolium and reduce the yellow tetrazolium within the gel to a blue precipitate. Since SOD inhibits this reaction, a colorless band indicates SOD activity [26].

**Figure 6:**
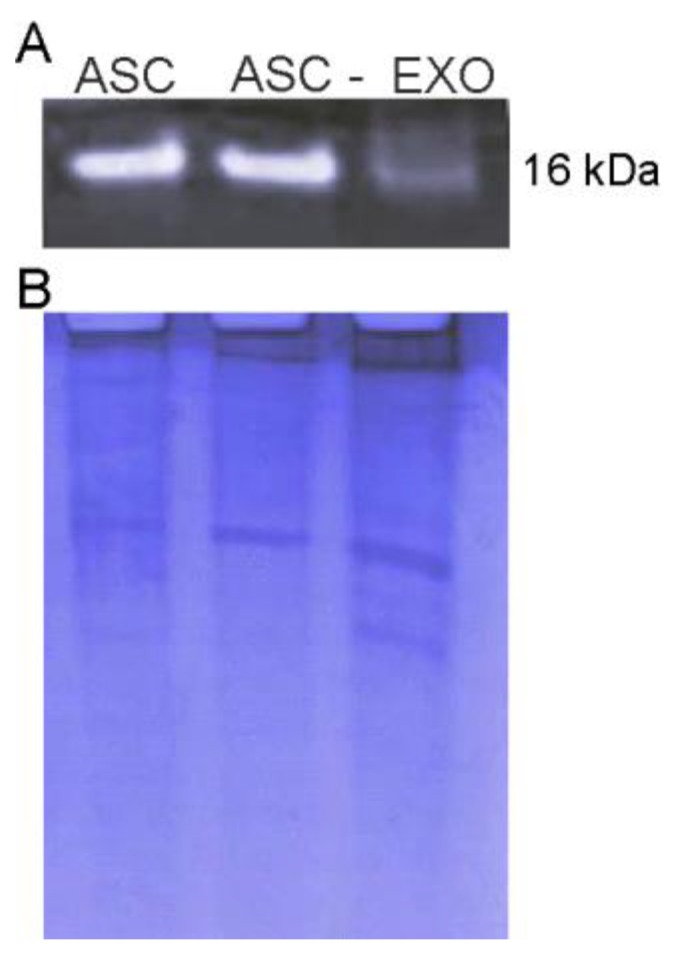
Zymogram assay of SOD1 protein. The assay shows that exosomes (EXO) contain the active form of SOD1 protein. The ASC and deprived ASC (ASC-) were used as the positive control (**A**). Comassie blue staining was used as total loading control (**B**).

As showed in Figure 6, SOD1 was found active both in ASC and exosomes, thus confirming the presence of active SOD1 in ASC-derived exosomes.

### 3.4. NSC-34(G93A) Cells Viability after ASC-Exosomes Treatment

**Figure 7:**
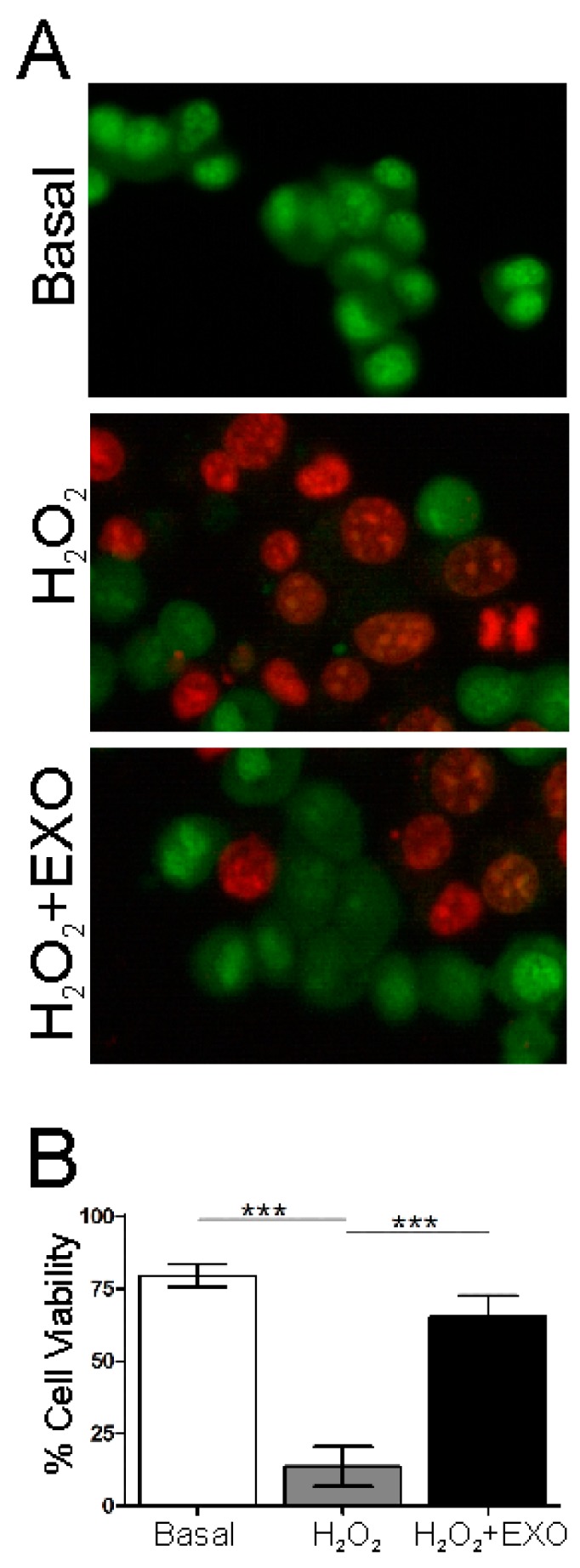
Acridine orange/propidium iodide (AO/PI) double staining on NSC-34(G93A) cells. Apoptotic and live cells were visualized after AO/PI double staining. The green fluorescence staining by AO indicate live cells, while orange/red fluorescence indicate the PI staining that bound to DNA after damaged membranes. The image shows cells in a basal condition (no cell death was detected and nucleus is uniformly distributed), cells after H_2_O_2_ treatment (the nucleus is located in bias and apoptosis-associated changes of cell membranes can be detected, indicating a process of disintegration) and cells after treatment with H_2_O_2_ and exosomes (H_2_O_2_ + EXO) in which a rescue of cells from death is detected, with an increase in cell viability compared to cells after H_2_O_2_ treatment Magnification 20× (**A**). The graph shows the percentage of cell viability of NSC-34(G93A) cells in basal condition and after H_2_O_2_ and ASC-exosomes treatment (H_2_O_2_ + EXO). Cell viability significant increased after ASC-exosomes treatment. One-way ANOVA and Bonferroni post-hoc analysis were performed between all the experimental conditions (*** *p* < 0.001) (**B**).

The beneficial effect of ASC-exosomes was evaluated on NSC-34(G93A) cells, confirming our previous data [14]. Cells were exposed to H_2_O_2_ to induce apoptosis [27] with or without the presence of ASC-exosomes. The exosomes neuroprotective effect was evaluated in terms of cell morphology, cell viability and apoptosis by cell counting after AO/PI double staining. The administration of ASC-exosomes in the culture medium determined a protective effect with an increase in cell viability, as detected after AO/PI double staining, where live cells showed green fluorescence and apoptotic cells showed orange/red fluorescence (Figure 7A). The H_2_O_2_ treatment determined a high cell death compared to the basal condition (with a mean of 13.94% of cell viability; *p* < 0.001), while the treatment with ASC-exosomes rescued the cells from apoptosis, with a significant increase in cell viability compared to the cells treated with H_2_O_2_ (mean of 64.98% of cell viability; *p* < 0.001) and restored cell viability with no significant difference compared to the control (basal condition, mean of 78.82% of cell viability) (Figure 7B).

### 3.5. Internalization of Exosomes-USPIO by NSC-34(G93A) Cells

To demonstrate that the effect on cell viability and the modulation of apoptotic pathway were induced by a biological activities of ASC-exosomes, the internalization of the vesicles by the cells was detected. To this purpose, the cells were incubated with exosomes-USPIO for 6 h, the time in which the neuroprotective effect was observed. Images of cells without exosomes incubation were used as the negative control (data not shown).

**Figure 8:**
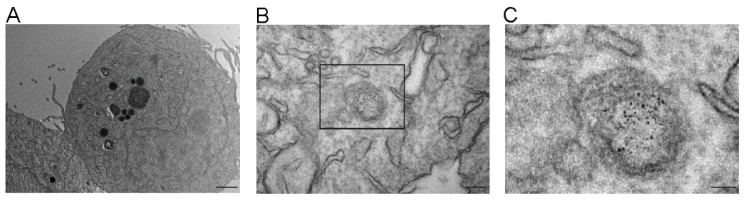
TEM images of cells treated with exosomes-ultra-small superparamagnetic iron oxide nanoparticles (USPIO). TEM images showed no damaged cell after ASC-exosomes treatment; magnification 4400×, scale bar 1 µm (**A**). In (**B**) note a representative image of phospholipidic membrane structure contained high electron-density particles, whose dimension are attributable to USPIO nanoparticles used to label ASC-exosomes; magnification 46,000×, scale bar 100 nm. In (**C**), a higher magnification of the section squared in (**B**) is shown; magnification 140,000×, scale bar 50 nm.

The cells visualized by TEM showed a morphology without alterations or damage after ASC-exosomes treatment (Figure 8A). Cells were characterized by a heterochromatic nucleus, cytoplasm with polyribosome, a high number of mitochondria and an expanded endoplasmic reticulum. In the cytoplasm were present phospholipidic membrane structures contained high electron-density particles, whose dimensions could be attributable to USPIO nanoparticles used to label ASC-exosomes (Figure 8B,C). The presence of these nanoparticles inside the cells suggested the intracellular internalization of exosomes content by the cells. Moreover, high electron-density particles were not found in the cytosol, in the nucleus or associated to the cell membrane.

### 3.6. Expression Profile of Apoptotic Markers in Exosome-Treated NSC-34(G93A) Cells

**Figure 9:**
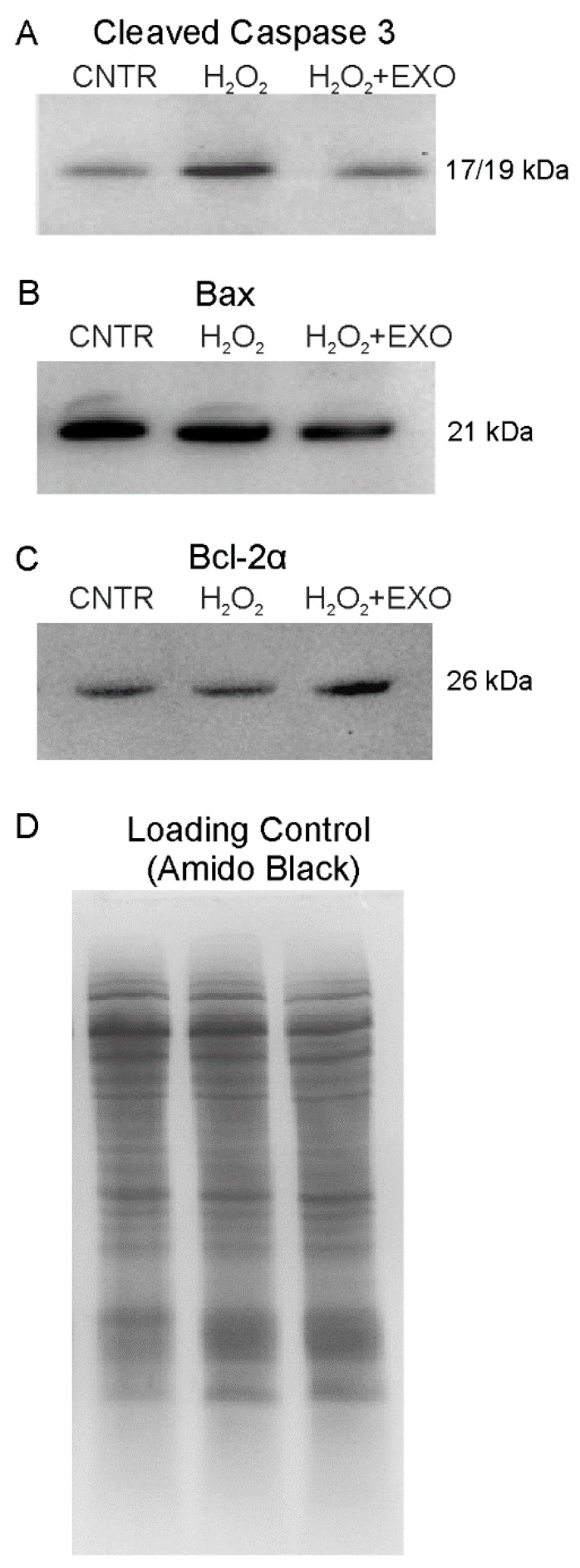
Expression profile of apoptotic markers in NSC-34(G93A) cells. The blots show western blot analysis of Cleaved Caspase 3 (**A**), Bax (**B**) and Bcl-2 α (**C**) proteins performed in NSC-34(G93A) cells (used as control, CNTR), NSC-34 (G93A) cells treated with H_2_O_2_ and NSC-34 (G93A) cells treated with H_2_O_2_ and exosomes (EXO). Amido Black staining was used as total loading control (**D**).

To investigate the neuroprotective potential of ASC-exosomes which was suggested by the identified proteins, the immunoblot analysis of some apoptotic markers was performed. In particular, we compared the expression level of the pro-apoptotic proteins cleaved-caspase 3 and Bax and of the anti-apoptotic protein Bcl-2 α in NSC-34(G93A) cells after 6h of exposure to H_2_O_2_ in the presence or absence of ASC-exosomes. As shown in Figure 9A, the expression level of Cleaved caspase 3 (~17/19 kDa) was increased after H_2_O_2_ cell treatment, compared to untreated cells. The two molecular weights indicate the fragments detected by the antibody (Cell Signaling: 9661), i.e., the large fragments (17/19 kDa) of activated caspase-3 resulting from cleavage adjacent to Asp175. Indeed, Caspase-3 activation occurs in two stages: First, caspase-3 pro-forms are cleaved by upstream caspases (such as active caspase-8) at Asp175 to generate intermediate heterotetramer complexes consisting of two p19 and two p12 peptides (p19/p12 complexes); then, the second stage involves removal of the short pro-domain from the p19 peptides by autocatalytic processing, and cleavage at residue Asp28, to generate the fully mature p17/p12 form of the enzyme [28].

On the other hand, the activated form of caspase-3 was reduced in cells treated with ASC-exosomes, compared to the cells treated with H_2_O_2_ and appeared similar to one of the untreated cells. In addition, we found Bax (~21 kDa) reduction in cells treated with exosome compared to H_2_O_2_ treated cells (Figure 9B). These data point out a protective role of exosomes in counteracting the expression of pro-apoptotic proteins. Concerning the anti-apoptotic protein Bcl-2 α (~26 kDa), the immunoblot results showed that it was increased in cells exposed to H_2_O_2_ in the presence of ASC-exosomes compared to cells without exosomes (Figure 9C).

Altogether these results underline the neuroprotective role of exosomes in counteracting a stress stimulus, giving an explanation of our previous published data in which we found an increase of cell survival after exosomes treatment compared to cells exposed to only H_2_O_2_ [14].

## 4. Discussion

The great interest of the scientific community in relation to the possible use of exosomes in a pathological condition is due to their role in intercellular communication [8]. Moreover, in the therapeutic application of stem cells, there is evidence that these cells exert their beneficial effect through the release of exosomes instead of their engraftment [7]. These extracellular vesicles, after their release, can be taken up from the other living cells, where they modify cell behavior. In recent years, several studies highlight the potential therapeutic use of exosomes in different diseases [4,29,30]. The effect of exosomes is strictly correlated to their content, that depends on the origin cells [29]. In view of a therapeutic application of exosomes, it is crucial to know the content of these vesicles to understand their mechanism of action. Moreover, this knowledge could open the way to manipulate the origin cells to obtain exosomes that are rich in the molecule directly implicated in the neuroprotective action.

Several studies report the proteomic content of extracellular vesicles isolated from different sources such as bone marrow MSC, umbilical cord, placenta, human ASC and human milk [31,32,33,34]. In our study we describe, for the first time, a comprehensive proteomic analysis of exosomes isolated from murine adipose stem cells. The ASC-exosomes proteomic profiling was performed to elucidate their neuroprotective mechanism of action pointed out in our previous data in an in vitro model of ALS [14] and this was confirmed in this study. We demonstrated that the presence of ASC-exosomes protects NSC-34 cells, transfected with different mutations of the human *SOD1* gene, from oxidative damage, increasing cell viability [14].

The proteomic analysis of ASC-exosomes revealed the presence of 189 exosomal proteins, that are mainly implicated in cellular pathways crucial to the protective effect of the vesicles, e.g., cell adhesion and negative regulation of the apoptotic process. As shown by the protein network and pathway analysis, this reveals the identity of the proteins and their interaction in a specific cellular pathway; the major proteins are implicated in response to stress and in the PI3K-Akt signaling pathway.

In ALS, oxidative stress and alteration of axonal transport are typical features of the disease pathogenesis [4]. In relation to this, the presence of proteins involved in the response to stress in ASC-exosomes, such as SOD1 and SOD3 are able to destroy free superoxide radicals and cytoskeleton proteins involved in cytoplasmic transport, and this could explain their protective effect on in vitro model of ALS. Moreover, the presence of *SOD1* could replace the enzymatic function of mutated *SOD1*, improving the response of ALS motoneurons to oxidative stress.

Besides, the presence of the ribonuclease RNase 4 in ASC-exosomes could be potential contributors to exosomes neuroprotection. The RNase 4 protein, displaying an angiogenic action, seems to be critical for ALS. In fact, the angiogenic, neurogenic and neuroprotective activities of RNase 4 have been recently demonstrated [35]. In addition, mutations in the RNase 4 gene were reported in ALS patients [36], confirming a potential use of ASC-exosomes as therapy in ALS.

Concerning the proteins involved in the PI3K-Akt signaling pathway, the analysis points out the presence of Igf1, the insulin-like growth factors. The Igf1 protein, by binding the receptor Igf1R, promotes cell proliferation and inhibits apoptosis by blocking the pro-apoptotic protein BAD and by phosphorylating apoptosis signal-regulating kinase 1 [37,38]. In relation to this, a study demonstrated that blocking the Igf1R and avoiding their binding to the Igf1 protein, an increase in pro-apoptotic proteins and a decrease in anti-apoptotic proteins, like Bcl-2 was observed, enhanced the apoptotic cascade [39].

Notably, Cytoscape/KEGG pathway analysis revealed also exosomal proteins implicated in the regulation of actin cytoskeleton, ECM-receptor interaction and focal adhesion. Recent findings suggested that cytoskeleton alteration contribute to motoneuron degeneration [40], and that either ECM-receptor interaction and focal adhesion are pathways in which are implicated the up-regulated genes of ALS patients [41]. In addition, ECM-receptor interaction was identified as reduced in iPSC-derived motor neurons of patients with C9ALS [42] and has been showed to affect motor neuron in ALS model mouse [43].

We demonstrated that exosomes induce a neuroprotective effect modulating the apoptotic pathway after their internalization by the cells. In particular, we observed a decrease of pro-apoptotic proteins, as cleaved-caspase 3 and Bax, and an increase of anti-apoptotic protein Bcl-2 α in cells treated with ASC-exosomes. The presence of proteins involved in the PI3K-Akt signaling pathway in ASC-exosomes, like Akt, could represent one of the possible mechanism of action by which these vesicles exert their effect in an in vitro ALS model.

Several studies point out that, in ALS patients as well as in the murine models of disease, the motoneuron death is due to the activation of the apoptotic pathway [44,45,46,47,48,49]. In the *SOD1*(*G93A*) murine model, the inhibition of caspase-1 and caspase-3 have a protective role in ALS, delaying the symptom progression and improving survival of animals [44]. Kaspar and colleagues report that the delivery of Igf1 in the same murine model prolongs life and delays the ALS progression through the PI3K-Akt pathway [47]. These evidences underline that an approach that counteracts the apoptotic pathway could be used as a possible therapy in ALS.

## 5. Conclusions

This study presents a comprehensive proteomic analysis of exosomes derived from murine ASC, indicating the possible mechanisms by which ASC-exosomes act. Since these extracellular vesicles contain proteins involved in several pathways that stimulate cell survival and proliferation, they represent a promising therapeutic approach in different neurodegenerative disease, including ALS. Studies conducted with an in vivo model of ALS will be necessary to confirm the possibility of using exosomes as therapy.

Moreover, this study provides the basis to ameliorate the efficacy of this possible non-cell based therapy. Indeed, the effect mediated by exosomes could be improved through the use of engineered ASC in order to obtain exosomes that contain a higher amount of the molecules responsible for their neuroprotective effect.

## Supplementary Materials

The following are available online at https://www.mdpi.com/2073-4409/8/9/1087/s1. Supplementary Material 1: Table S1. Identified proteins. A total of 189 proteins with a peptide confidence cut-off of 99% (FDR < 1%) were identified in ASC-exosomes. Supplementary Material 2: Table S2. Transmembrane domains of the identified proteins predicted using the TMHMM program. ExpAA: expected number of amino acids in transmembrane helices. Numbers of TDMs: Number or transmembrane domains. Supplementary Material 3: Table S3. GO enrichment analysis of the 189 ASC-exosomal proteins using DAVID software. GOBPs (A) and GOMFs (B) and GOCCs (C) represented by the ASC-exosomal proteins. The ‘Count’ represents the number of proteins with the corresponding GOBPs, GOMFs or GOCCs. The *p*-values imply the significance of being enriched by the exosomal proteins. The percentage represents the portion of the genes encoding the proteins with the corresponding GOBPs, GOMFs or GOCCs in the 189 ASC-exosomal proteins. Supplementary Material 4: Table S4. KEGG enriched pathways analysis obtained using ClueGO app for Cytoscape. ClueGo analysis was performed by setting Bonferroni step down correction (adjusted group *p*-value < 0.05).
